# Signal and regulatory mechanisms involved in spore development of *Phytophthora* and *Peronophythora*

**DOI:** 10.3389/fmicb.2022.984672

**Published:** 2022-09-09

**Authors:** Junjian Situ, Pinggen Xi, Long Lin, Weixiong Huang, Yu Song, Zide Jiang, Guanghui Kong

**Affiliations:** ^1^Department of Plant Pathology, Guangdong Province Key Laboratory of Microbial Signals and Disease Control, South China Agricultural University, Guangzhou, China; ^2^Department of Plant Pathology, Nanjing Agricultural University, Nanjing, China

**Keywords:** oomycete, zoospore, oospore, zoosporogenesis, sporulation

## Abstract

Oomycetes cause hundreds of destructive plant diseases, threatening agricultural production and food security. These fungus-like eukaryotes show multiple sporulation pattern including the production of sporangium, zoospore, chlamydospore and oospore, which are critical for their survival, dispersal and infection on hosts. Recently, genomic and genetic technologies have greatly promoted the study of molecular mechanism of sporulation in the genus *Phytophthora* and *Peronophythora*. In this paper, we characterize the types of asexual and sexual spores and review latest progress of these two genera. We summarize the genes encoding G protein, mitogen-activated protein kinase (MAPK) cascade, transcription factors, RNA-binding protein, autophagy-related proteins and so on, which function in the processes of sporangium production and cleavage, zoospore behaviors and oospore formation. Meanwhile, various molecular, chemical and electrical stimuli in zoospore behaviors are also discussed. Finally, with the molecular mechanism of sporulation in *Phytophthora* and *Peronophythora* is gradually being revealed, we propose some thoughts for the further research and provide the alternative strategy for plant protection against phytopathogenic oomycetes.

## Introduction

**Figure 1:**
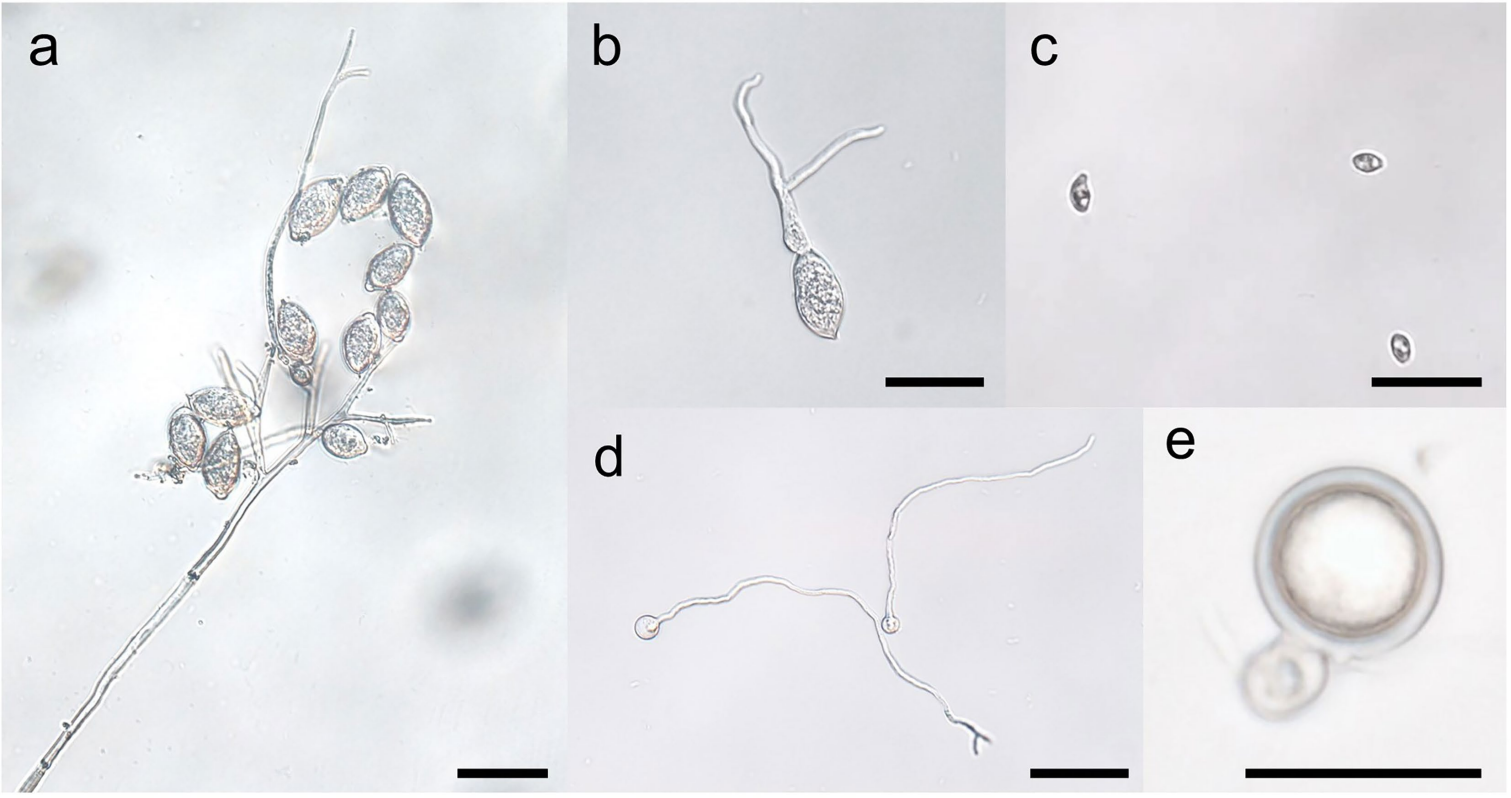
Disease symptoms of *Peronophythora litchii* infection. **(A–C)** Symptoms of *P. litchii* on litchi fruit, tender leaves and panicles. **(D)** Mature sporangiophores and sporangia of *P. litchii* on the infected tissue. Bar represents 100 μm.

**Figure 2:**
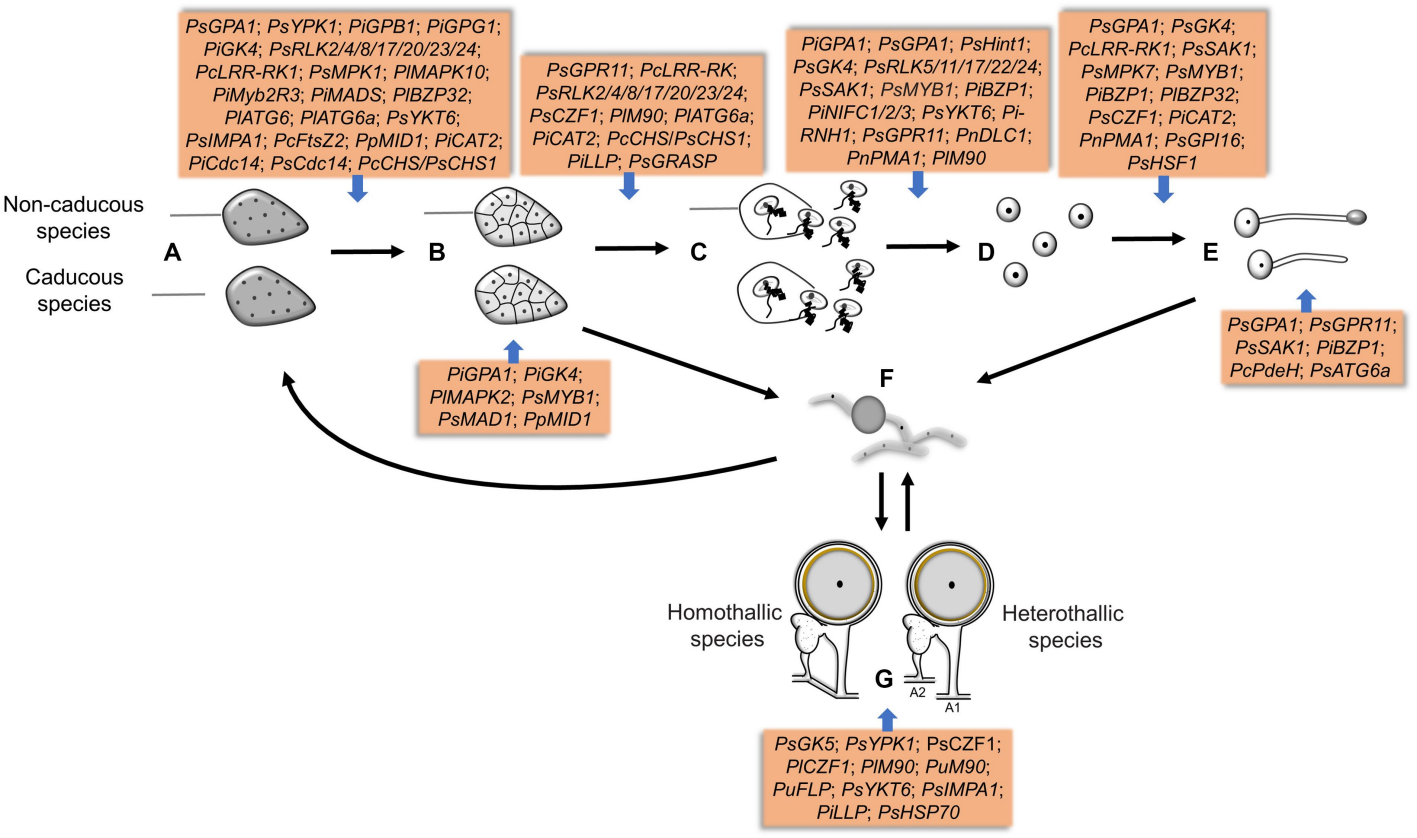
Disease cycle of *Peronophythora litchii* on host plant. The cycle can occur on leaves and fruit in the stage of litchi growth and fruit storage.

As a class of eukaryotic microorganisms, oomycetes show a similar life cycle with filamentous fungi; however oomycetes are evolutionarily close to photosynthetic algae and distant from true fungi (Tyler et al., 2006; Cavalier-Smith et al., 2018). Among oomycetes, *Phytophthora* and *Peronophythora* contain a large number of destructive plant pathogens, which seriously threaten agriculture, forestry and ecosystem. An example of this is the late blight pathogen *Phytophthora infestans* which is notorious for severely damaging the European potato industry in 1840 and leading to Ireland famine (Kamoun et al., 2015). Nowadays, late blight remains a major problem around the world with global yield losses estimated at more than £10 billion a year (Leesutthiphonchai et al., 2018a). *Phytophthora capsici* and *Phytophthora sojae* severely damage cucurbits and soybean, respectively (Kamoun et al., 2015). Litchi downy blight caused by *Peronophythora litchii* is the most destructive disease in litchi (Kong et al., 2021; Figure 1). *P*. *litchii* is evolutionarily closed to *Phytophthora* species, and the life cycle of *P. litchii* is illustrated in Figure 2. Therefore, *Phytophthora* and *Peronophythora* are reviewed together in this paper.

The *Phytophthora* and *Peronophythora* species are both hemibiotrophic microorganisms, which plays a notable role in scientific research. The research on potato late blight initiated the science of plant pathology. However, the classical genetic and molecular studies of oomycete lag behind true fungi. Several true fungi, such as *Aspergillus nidulans*, *Magnaporthe oryzae*, and yeasts were used as models for studying spore biology (Ebbole, 2007; Neiman, 2011; Etxebeste and Espeso, 2020), but knowledge of fungal development had limited the relevance to oomycete, because they belong to different kingdoms and produce different types of spores (Judelson and Blanco, 2005).

Until the 1990s, effective genetic tools began to be developed for oomycete, which promoted the progress of its biological research including spore development. DNA-mediated transformation was successfully developed for *P. infestans*, making it a model for the genus (Judelson et al., 1991). Subsequently, the establishment of gene-silencing technology enabled gene function to be analyzed (Kamoun et al., 1998; van West et al., 1999a; Whisson et al., 2005). Both GFP and GUS were used successfully as reporter genes in *Phytophthora*, which facilitated the transcription and subcellular localization analysis (van West et al., 1999b). And recent CRISPR/Case9-mediated genome editing technology has greatly accelerated the process of functional genomics of oomycete (Fang and Tyler, 2016; Situ et al., 2020; Ah-Fong et al., 2021). Moreover, the advances in sequencing technologies in the last two decades rapidly expanded the available genome and transcriptional data of *Phytophthora* and *Peronophythora* species (Ye et al., 2016; Ah-Fong et al., 2017; McGowan and Fitzpatrick, 2020). Since the first publication of oomycete genome sequences of *P. ramorum* and *P. sojae* in 2006 (Tyler et al., 2006), more than 27 *Phytophthora* species genome have been available (McGowan and Fitzpatrick, 2020), which has promoted the research of molecular mechanism of development and pathogenicity in oomycete.

Given that the spores play a critical role in plant-oomycete interaction, and that spore formation mechanisms have advanced significantly in the last decades, it is time to systematically summarize these findings and discuss the potential modulation of sporulation in plant disease management. In this review, the focus will be on the recent research progress with respect to formation and germination of sexual and asexual spores in *Phytophthora* and *Peronophythora*, especially those findings that have been proved by genetic strategies.

## Asexual and sexual spores of *Phytophthora* and *Peronophythora*

The asexual spores of *Phytophthora* and *Peronophythora* include sporangia (or zoosporangia), zoospores and chlamydospores. Sporangia are multinucleate and release zoospores in aqueous environment (Judelson and Blanco, 2005). Zoospores are single nucleated and wall-less cells that can swim with the help of two flagella; one tinsel (or anterior) flagellum, and one whiplash (or posterior) flagellum (Walker and van West, 2007). The thick-walled resting chlamydospores could be produced by some *Phytophthora* species but not *P. infestans* and *P. litchii*. Besides, the sexual oospores display double walls and contain one or more pellucid bodies (nuclei) and a well-defined ooplast (Boutet et al., 2010).

## Sporangia develop at the termini of sporangiophore

**Figure 3:**
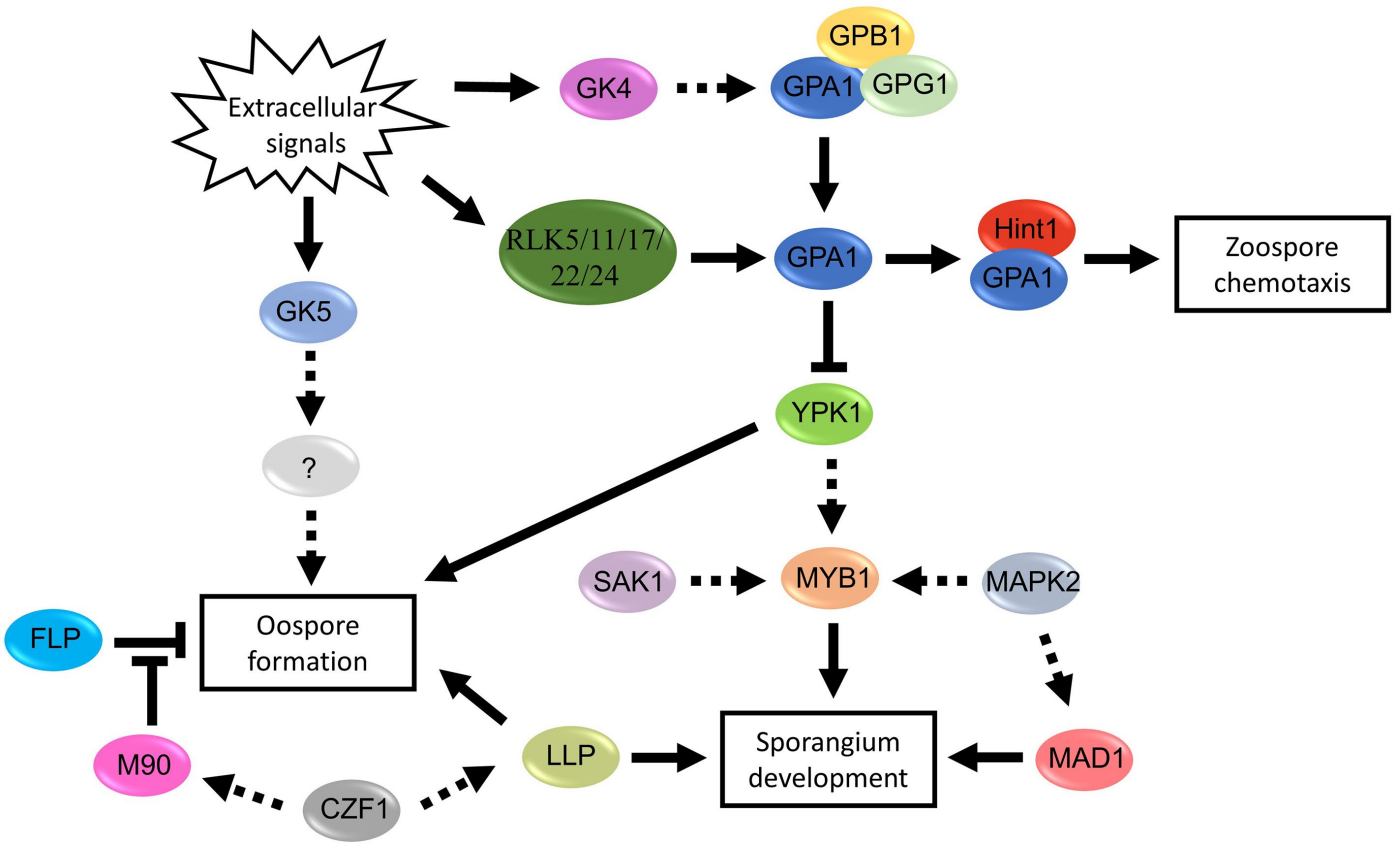
Stages of the spore cycles of *Peronophythora litchii*. **A–E** represent sporangiophore and sporangia, sporangium germination, zoospores, cysts germination, mature oospore, respectively. Bar represents 50 μm.

Compared with oospores, the yield of sporangia is very huge in *Phytophthora* and *Peronophythora*. Sporangia develop at the termini of sporangiophore (Figure 3). Sporangiophore is often branched and each terminus bears one sporangium in *Phytophthora* and *Peronophythora* (Judelson and Blanco, 2005). In *P. litchii*, a new sporangiophore could develop from mycelium or a terminus of another sporangiophore, the late process could be repeated limited times resulting multideterminate in tiers of sporangiophores, each with its own branching system (Figure 3). In *P. litchii*, PlATG6a positively regulates the branch formation of sporangiophores, while PlBZP32 exhibits the opposite effect (Kong et al., 2020; Wang et al., 2022). When sporangium matures, an apical papilla forms in most species and a basal septum develops. In caducous species such as *P. infestans* and *P. litchii*, water or wind can separate the sporangium from the sporangiophore and spread it for several kilometers (Aylor, 2003). This separation of sporangia is convenient for researchers as the sporangia can be easily purified for analysis. By contrast, the sporangia of non-caducous species such as *P. sojae* remain attached to the sporangiophores in water. Therefore, it is not easy to purify the sporangia of *P. sojae*, but zoospores could be collected for research. Besides, little effort has been made to decipher the underlying mechanism of the distinctive pattern of sporangiophores formation and sporangia caducity in *Phytophthora* and *Peronophythora* species so far, even though it could be used for crop protection.

## Sporangia and zoospores are the main inocula of *Phytophthora* and *Peronophythora*

Asexual sporangia and zoospores are the main inocula of *Phytophthora* and *Peronophythora*. Sporangia have a remarkable ability to germinate in two different ways: direct germination and indirect germination (also known as zoosporogenesis), which is depended on the ambient temperature. Low temperature could promote the release of zoospores from sporangia. Both germination ways require the sporangia to be immersed in aqueous environment. Germination by the zoosporogenesis is believed to be important in disease. The biflagellate zoospores swim after release. The anterior flagellum is probably responsible for pulling the zoospore through the water whereas the posterior flagellum acts as a rudder for steering the cell (Walker and van West, 2007). Once a suitable site has been found, the zoospores encyst and become immobilized. The flagella are either discarded or retracted into the cell depending on the species (Hardham and Hyde, 1997). The encystment process involves the rapid cell wall formation. The dual germination strategy increases the possibility of *Phytophthora* sporangia to colonize plants in diverse environments. However, the genes involved in the sporangium germination pathways of *Phytophthora* and *Peronophythora* are largely unknown.

## Pathways involved in sporangium formation and cleavage

The heterotrimeric G-protein pathway regulates cellular responses to a wide range of extracellular signals in all eukaryotes (Xue et al., 2008). The sporangium formation and cleavage are also stimulated by environment to a great extent (Xue et al., 2008), the heterotrimeric G-protein pathway therefore is thought to be associated with the sporulation. In *Phytophthora* spp., G-protein have only one Gα, one Gβ, and one Gγ subunit (Qiu et al., 2020). The G protein α subunit GPA1 directly interacts with serine/threonine protein kinase YPK1 and prevents nuclear localization of YPK1, which leads to suppression of sporangia formation in *P. sojae* (Qiu et al., 2020). In the previous studies, the Gβ and Gγ (PiGPB1 and PiGPG1) of *P. infestans* were also demonstrated to be crucial for sporangium development and production (Latijnhouwers and Govers, 2003; van den Hoogen et al., 2018). The G-protein signaling normally initiates from membrane-bound G-protein coupled receptors (GPCRs) that receive extracellular signals and then activate the Gα subunit (Rosenbaum et al., 2009). Genome analysis revealed that *Phytophthora* spp. have over 60 GPCR genes, among which several are fused with a C-terminal phosphatidylinositol phosphate kinase (PIPK) domain (Bakthavatsalam et al., 2006). The full set of GPCR-PIPKs (GKs) of *P. infestans* was identified in 2013 and subsequent analysis proved that the overexpression of PiGK4 produced sporangia with aberrant cell walls, aberrant germination of sporangia and defects in cytoplasmic cleavage and the release of zoospores (Hua et al., 2013).

Another type of signal receptor leucine-rich repeat receptor-like kinases (LRR-RLKs) are also involved in *Phytophthora* spore development. The *P. capsici PcLRR*-*RK1*-silenced mutants exhibited abnormal shape and size of sporangia and lower production of sporangia and zoospores (Safdar et al., 2017). The systematical characterization of all 24 LRR-RLK genes (PsRLKs) from *P. sojae* revealed that PsRLK2/4/8/20/23 positively regulate the production of sporangia and zoospores, whereas PsRLK17/24 negatively regulate these processes (Si et al., 2021b).

In oomycetes and fungi, MAPK cascades also play a key role in the development and plant infection (Jiang et al., 2017; Gonzalez-Rubio et al., 2019). Sporangium formation is severely diminished in *PsMPK1*-silenced mutants (Li et al., 2014). MAPK10 also positively regulates the production of sporangia in *P. litchii*. *P. litchii* mitogen-activated protein kinase MAPK2 is essential for the sporangial cleavage of *P. litchii* (Huang et al., 2021). And the transcription of *PlMYB1* and *PlMAD1* (homologs of *PsMYB1* and *PsMAD1*; described later) are downregulated in *PlMAPK2* mutants (Huang et al., 2021).

Following the signal transduction pathways, transcription factors are the next players for controlling sporangium formation and germination. The *P. sojae* MYB transcription factor MYB1, regulated by PsSAK1, controls the differentiation of sporangial cytoplasm and nuclei rearrangement, which finally affects the sporangia cleavage during zoospores release (Zhang et al., 2012a). Eight Myb transcription factors are upregulated during sporulation and silencing of *PiMyb2R3* results in suppressing sporulation in *P. infestans* (Xiang and Judelson, 2010, 2014). A basic leucine zipper (bZIP) transcription factor PlBZP32 is negatively associated with sporangia production (Kong et al., 2020). *P. sojae* MADS-box transcription factors PsMAD1 also affects the zoosporogenesis similarly to PsMYB1 (Lin et al., 2018). Silencing of *PiMADS* severely impairs sporangium production (Leesutthiphonchai and Judelson, 2018b). The transcriptome of *PiMADS*-silenced mutant also provides new data for identifying more genes involved in sporangium formation and revealing the network (Leesutthiphonchai and Judelson, 2018b).

Autophagy is a conserved cellular process allowing the organisms to recycle their intracellular components by sequestrating the cytosolic macromolecules or used organelles within the autophagosomes and delivering the contents to the lysosomes or vacuoles (Feng et al., 2014). Twenty-six genes in the core autophagy machinery were identified in *P. sojae* genome and silencing of *PsAtg6a* led to reduce sporangium production (Chen et al., 2017). In *P. litchii*, knockout of *PlATG6a* significantly impaired the pathogen sporangium production but promoted the release of zoospores (Wang et al., 2022). Further study is need to reveal the mechanism of ATG genes involved in asexual reproduction in oomycete.

Additionally, cell cycle protein PiCdc14/PsCdc14, importin α PsIMPA1 and FtsZ protein PcFtsZ2 are also involved in sporangia production (Ah-Fong and Judelson, 2003; Zhao et al., 2011a; Yang et al., 2015; Li et al., 2020b). Golgi reassembly stacking protein PsGRASP is associated with zoosporogenesis (Si et al., 2021a). Soluble N-ethylmaleimide-sensitive factor attachment protein receptor PsYKT6, Mating pheromone-induced death 1 PpMID1, catalase PiCAT2 and two chitin synthase PcCHS/PsCHS1 regulate both processes (Zhao et al., 2011b; Hwu et al., 2017; Cheng et al., 2019; Wang et al., 2020). The above studies identify the pathways from membrane receptor, signal transduction to the downstream transcriptional regulation involved in sporangium formation and cleavage. Nevertheless, the relationship between these components is rarely known, therefore, the regulatory network of how sporangia perceive environment temperature and determine the germination type remains to be further explored.

## Regulators of zoospore motility, chemotaxis and encystment

After zoospore release, Ca^2+^ helps to regulate their swimming and encystment, phosphatidic acid might also be involved in zoospore motility and encystment (Hardham and Hyde, 1997; Tani et al., 2004). Through study of the effect of inhibitors on zoospore development in *P. infestans*, a putative protein kinase gene induced during zoosporogenesis that encoded a protein resembling Ca^2+^ and calmodulin-regulated serine/threonine protein kinase was identified (Judelson and Roberts, 2002). The mRNA accumulation of this protein kinase is first detected soon after chilling sporangia in water and persisted in motile zoospores and germinated cysts, but not in other tissues (Judelson and Roberts, 2002).

The important roles of zoospore motility for successful infection have been shown through silencing of *PiGPA1* and *Pibzp1* (Latijnhouwers et al., 2004; Blanco and Judelson, 2005). Moreover, *PsGPR11* and *PsSAK1* are involved in the regulation of swimming time (Li et al., 2010; Wang et al., 2010). Further research suggested that some aberrant swimming behaviors are probably related to defects in flagella composition. The study of DEAD-box RNA helicase gene *Pi-RNH1* showed that *Pi-RNH1*-silenced lines released larger zoospores which were observed to have multiple flagella, low tolerance to osmotic pressure and contained several large vesicles with smaller lipid bodies fused to them (Walker et al., 2008). On the contrary, the *Phytophthora parasitica* dynein light chain 1 (DLC1), one of the flagellar axoneme component, positively regulated flagella formation and zoospore motility (Narayan et al., 2010). *PnPMA1*, encoding an atypical plasma membrane H^+^-ATPase, also regulated the formation of zoospore flagellate in *P. parasitica*. High levels of *PnPMA1* silencing resulted in production of non-flagellate and large aberrant zoospores, rapid transition from zoospores to cysts (Zhang et al., 2012b).

Chemotaxis is another important feature for zoospore to initiate infection in the disease cycle (Hua et al., 2015). The G protein α subunit is first shown to regulate the chemotaxis of *P. infestans* zoospores (Latijnhouwers et al., 2004). Further studies found that the histidine triad (HIT) domain-containing protein PsHint1 is associated with PsGPA1 and co-regulates zoospore chemotaxis to isoflavone and encystment (Hua et al., 2008; Zhang et al., 2016). This study also indicated that chemotaxis and motility are controlled by two different pathways, both of which are regulated by the Gα subunit, while Hint1 is only involved in the former. Yang et al. reported that the GPCR-PIPK PsGK4 was involved in zoospore chemotaxis towards isoflavone as well as soybean roots (Yang et al., 2013). Recently, LRR-RLKs are found to act on zoospore chemotaxis (Si et al., 2021b). Absence of LRR-RLK encoding genes *PsRLK5/11/17/22/24* results in severe defects in recognition of isoflavones by *P. sojae* zoospore (Si et al., 2021b). Interestingly, these five PsRLKs are demonstrated to regulate this process by direct interacting with PsGPA1 *via* their intracellular kinase domains (Si et al., 2021b). Whether there is a functional relationship between PsHint1 and these membrane-localized kinases in the signal pathway of chemotaxis deserve to be investigated.

## Signals in zoospore–zoospore interactions

Recognition of the host by zoospores is often referred to a variety of signals, for example chemical, electrical field and physical feature from host (Tyler, 2002; van West et al., 2002; Hua et al., 2015; Bassani et al., 2020a). Recent experimental evidence has demonstrated that zoospore-zoospore interactions can lead to “pattern swimming” in the absence of chemical or electrical signals from plants. Zoospore-free fluid (ZFF) prepared from zoospore suspension stimulates cyst germination and induces a tactic response to enhance zoospore auto-aggregation and infection (Kong and Hong, 2010a; Kong et al., 2010b). Another study in *P*. *parasitica* showed that the perception of a K^+^ gradient induces zoospore coordinated motion and aggregation, which produce vesicular and fibrillary material discharged at cell-to-cell communication (Bassani et al., 2020b). Leucine is also a signaling molecule secreted from zoospore and could regulate zoospore germination and infection by *P. erythroseptica* (Jiang et al., 2019). These zoospore–zoospore communication and coordinated behavior is analogous to bacterial quorum sensing. Additionally, a diurnal effect has been observed for production of propagules in *P. ramorum*. Large differences in sporangium and zoospore numbers were observed for the dark versus light periods (Tooley et al., 2020). However, it is not yet clear how these signals are generated in local soil or water niches and which genes govern the sensing and subsequent responses of zoospore.

## Molecular basis of cyst germination and appressorium formation

The cyst development, especially germination and the formation of infectious hyphae and appressorium that penetrate the host cells is the key step for infection. In a previous study, co-silencing of three nuclear LIM interactor-interacting factors encoded genes (*NIFC1*, *NIFC2* and *NIFC3*) in *P. infestans* impaired cyst germination (Judelson and Tani, 2007). Subsequently, more positive regulators of cyst germination were found in *P. sojae*, for example, the GPA1, Hint1, GPR11, SAK1 and HSF1 (Hua et al., 2008; Li et al., 2010; Wang et al., 2010; Sheng et al., 2015; Zhang et al., 2016). LRR-RK1 and BZP32 are also involved in cyst germination in *P. capsici* and *P. litchii*, respectively (Safdar et al., 2017; Kong et al., 2020). Recently, the asparagine (Asn, N)-linked glycosylation, a ubiquitously distributed post-translational modification (Nagashima et al., 2018), is reported to participate in this process (Zhang et al., 2021). Site-directed mutagenesis in the N-glycosylation site of the GPI transamidase component protein (GPI16) and heat shock protein 70 (HSP70) make *P. sojae* cyst defeat in germination (Zhang et al., 2021).

Other than germination, abnormal growth of the germ tube can lead to failure of zoospore infection. For instance, silencing of *PsMPK7* resulted in abnormal germinated cysts apical swelling, which might be due to the disturbance of polarized growth (Gao et al., 2015). Later, a study of the *P. capsici* high-affinity cAMP phosphodiesterase further confirmed the significance of polarized growth in cyst germ tube (Li et al., 2020a,b). The formation of appressorium is the last step of zoospore infection. Nevertheless, the regulators in this process are still largely unknown. Only previous research of *Pibzp1* showed that this gene silenced mutants failed to develop appressoria and were unable to infect plants (Blanco and Judelson, 2005). Appressorium attachment and penetration of the plant cuticle and cell wall are the key processes for successful colonization of pathogens, given that the mechanism of appressorium development deserves more attention in the future research.

## Mating strategies and oospore formation

Oospores are important inocula in the next growing season as they can survive in soil or debris for several years, particularly for the homothallic (self-fertile) species. Oospores are also significant for heterothallics when both mating types (A1 and A2) share the same geographical space. In both homo- and hetero-thallics, male and female gametangia can develop and then fuse to form oospores. The germinated oospores produce either a hyphal tube, which can directly infect plants, or a germ sporangium, which acts like an asexual sporangium. Eight mating-induced genes were identified by suppression subtractive hybridization (Fabritius et al., 2002); among them, M90 encodes a member of the Puf family of translational regulators and highly expresses in sexual and asexual sporulation structures in *P. infestans* (Cvitanich and Judelson, 2003). Recently, researchers have confirmed that M90 is critical for the oospore formation in *P. litchii* and *Pythium ultimum* by gene silencing and CRISPR/Cas9-mediated genome editing technology (Jiang et al., 2017; Feng et al., 2021). A tripartite recognition motif (TRM) in the Puf domain of PuM90 could bind to the 3′– untranslated region (UTR) of PuFLP, and thereby repress PuFLP mRNA level to facilitate oospore formation. Considering that M90 is conserved in oomycete, its homologs may function *via* similar mechanism. A study of the *P*. *infestans* loricrin-like protein *via* scanning electron microscopy (SEM) found that many sunken areas were observed on the surfaces of the oogonia of *PiLLP*-silenced transformants, which indicated the loss of turgor in oogonia. The Nile red staining and fluorescence microscopy observation further found that red fluorescence-emitting substance was scattered instead of aggregating into a red ball in *PiLLP*-silenced transformants, thus, the sexual development blocked in the stage of oospore wall formation (Guo et al., 2017). Moreover, the C_2_H_2_ zinc finger protein CZF1 has been demonstrated to be involved in oospore development in *P. sojae* and *P. litchii* (Wang et al., 2009; Zhu et al., 2022). Interestingly, the transcription level of *M90* and *LLP* are downregulated in *CZF1* knockout mutant, suggesting that *CZF1* may control the transcription of these two genes. Additionally, YPK1, YKT6, GK5, IMPA1 and N-glycosylation in HSP70 also play an important role in oospore production in *P. sojae* (Zhao et al., 2011b; Yang et al., 2013, 2015; Qiu et al., 2020; Zhang et al., 2021). Although above studies described the genes involved in oospore formation, how these genes relate to the mechanism of oospore formation remains unclear. Secretion of hormones is the prerequisite of sexual propagation. Once a heterothallic is stimulated by the hormone from opposite mating type, the hybrid and selfed oospores can be formed, while most homothallics produce both (Ko, 1988; Judelson, 1997). The chemical basis of *Phytophthora* mating hormone was first proposed in *P. parasitica* (Qi et al., 2005). We speculate that PsGK5 may be involved in the perception of these mating signal. How these genes are involved in synthesis and/or response to the mating hormones needs to be further explored.

## Conclusion and further research

**Figure 4:**
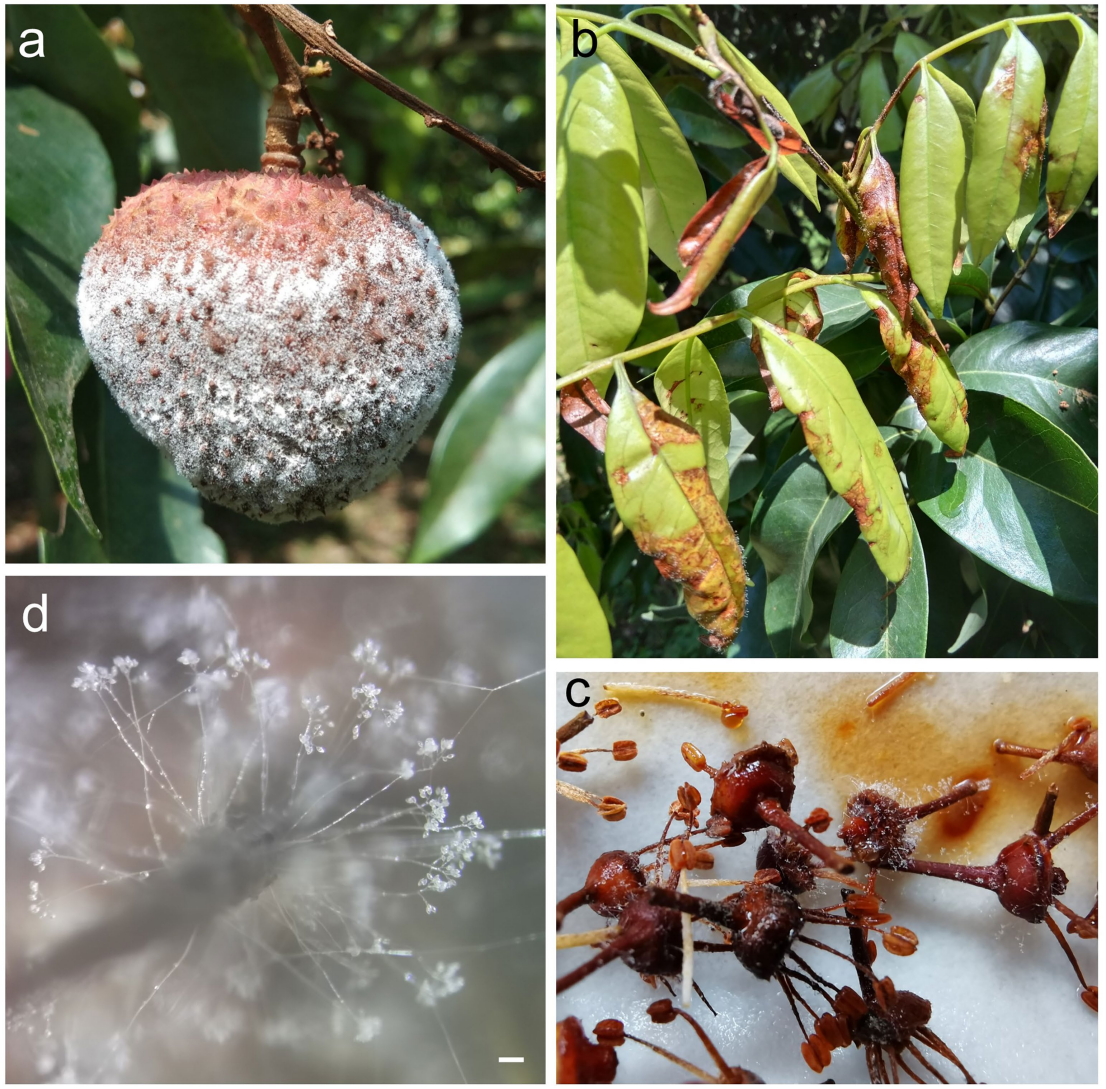
The genes involved in different sporulation and germination stages in *Phytophthora* and *Peronophythora*. **A–G** represent sporangia, sporangia cleavage, zoospores release, cysts, cysts germination, mycelia chlamydospores and oospores, respectively.

**Figure 5:**
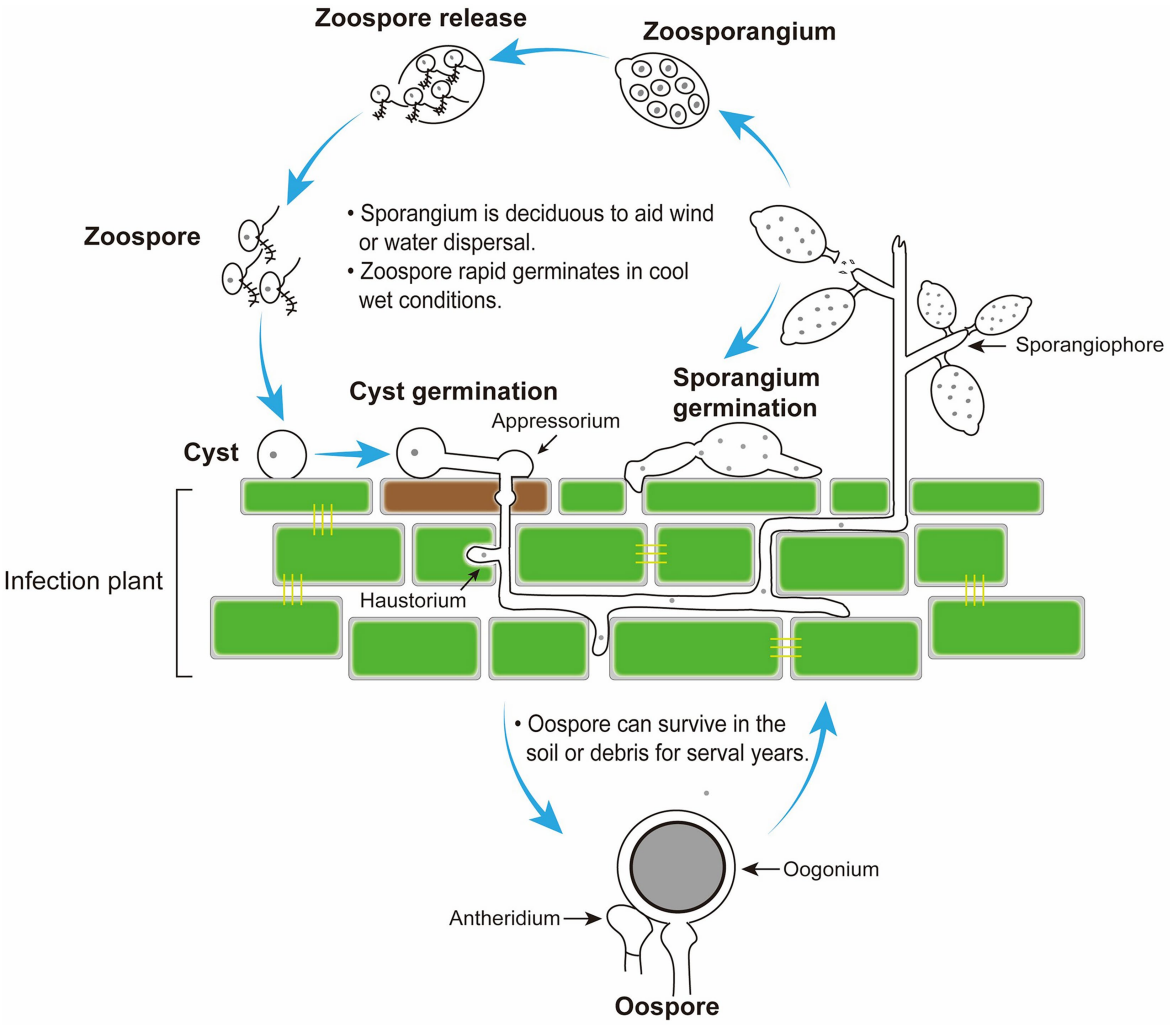
Proposed signal pathways involved in spore development of *Phytophthora* and *Peronophythora*. Arrows with solid line represent direct positive regulation, arrows with dotted line represent potential positive regulation and T-line represent negative regulation.

Genetic manipulation and omics have greatly promoted the progress of molecular biology of oomycete spores in the last decades. This paper reviewed the signal and regulatory mechanism of spore development in the *Phytophthora* and *Peronophythora*, and aimed for better understanding why they cause such destructive and persistent diseases. Benefiting from the advance in molecular biology, more and more sporulation-related genes have now been characterized (Figure 4; Table 1); therefore, we draw a portrait of the expansive knowledge. G-protein pathway, MAPK cascades and transcription factors are involved in the perception of extracellular signal and the network of spore formation. Here, we propose the relationship of the main regulators in *Phytophthora* and *Peronophythora* spore development (Figure 5). It is obviously that the G protein α subunit GPA1 play a central role for asexual spore development. While, for sexual spore formation, more works can be done around the downstream signal transduction components of GK5 and mRNAs targeted by M90. Excitingly, in regard to this plethora of arenas, much remains to be investigated and understood. Researchers might use yeast-two hybrid, RIP or ChIP-seq technology to identify more direct interaction elements of these spore formation-related proteins and characterize their regulatory networks. Additionally, it is worth studying the formation of appressorium and haustorium from the geminated cyst, because they are also important weapons for these plant destroyers.

**Table 1:** Sporulation-related genes discussed in this review.

Gene	Annotation	Function/phenotype	References
*PiGPA1*	G protein α subunit	Sporangium cleavage, zoospore swimming behavior and chemotaxis, aprpressorium formation, pathogenicity	Latijnhouwers et al. (2004)
*PiGPB1*	G protein β subunit	Mycelial growth, sporangium production and morphology, pathogenicity	Latijnhouwers and Govers (2003)
*PiGPG1*	G protein γ subunit	Mycelial growth, sporangium production and morphology	van den Hoogen et al. (2018)
*PsGPA1*	G protein α subunit	Sporangium production, zoospore chemotaxis and encystment, cyst germination, pathogenicity	Hua et al. (2008) Qiu et al. (2020)
*PsYPK1*	Serine/threonine protein kinase	Mycelial growth, sporangium production, oospores production, pathogenicity	Qiu et al. (2020)
*PiGK4*	G-protein-coupled receptor with a phosphatidylinositol phosphate kinase domain	Sporangium morphology and germ tube development, sporangium cleavage, pathogenicity	Hua et al. (2013)
*PsGK4*	G-protein-coupled receptor with a phosphatidylinositol phosphate kinase domain	Zoospore chemotaxis and encystment, cyst germination, pathogenicity	Yang et al. (2013)
*PsGK5*	G-protein-coupled receptor with a phosphatidylinositol phosphate kinase domain	Oospore production, pathogenicity	Yang et al. (2013)
*PsGPR11*	G-protein-coupled receptor	Zoospore production and swimming behavior, cyst germination, pathogenicity	Wang et al. (2010)
*PcLRR-RK1*	Leucine-rich repeat receptor-like kinase	Mycelial growth, sporangium production and morphology, zoospore production, cyst germination, pathogenicity	Safdar et al. (2017)
*PsRLK2/4/5/8/10/11/12/15/16/17/18/19/20/21/23/24*	Leucine-rich repeat receptor-like kinase	Responses to oomycetecides and bacteria antagonists, zoospore production and chemotaxis, pathogenicity	Si et al. (2021b)
*PsSAK1*	Mitogen-activated protein kinase	Zoospore swimming behavior, cyst germination and aprpressorium formation, salt stresses tolerance	Li et al. (2010)
*PsMPK1*	Mitogen-activated protein kinase	Mycelial growth, sporangium production, cell wall integrity, pathogenicity	Li et al. (2014)
*PsMPK7*	Mitogen-activated protein kinase	Cyst germination, oospore production, extracellular laccase activity, oxidative and osmotic as well as salt stress tolerance, reactive oxygen species detoxification, pathogenicity	Gao et al. (2015)
*PlMAPK10*	Mitogen-activated protein kinase	Mycelial growth, sporangium production, pathogenicity, extracellular laccase activity	Jiang et al. (2018)
*PlMAPK2*	Mitogen-activated protein kinase	Sporangium cleavage, pathogenicity, extracellular laccase activity,	Huang et al. (2021)
*PsMYB1*	R2R3-type Myb transcription factor	Sporangium cleavage, zoospore swimming behavior and encystment, cyst germination, pathogenicity	Zhang et al. (2012a)
*PiMyb2R1/* *PiMyb2R3/* *PiMyb2R4/* *Myb3R6*	R2R3/R1R2R3-type Myb transcription factor	Mycelial growth, sporangium production and germination	Xiang and Judelson (2010) Xiang and Judelson (2014)
*PsMAD1*	MADS-box transcription factor	Sporangium cleavage, pathogenicity	Lin et al. (2018)
*PiMADS*	MADS-box transcription factor	Sporangium production	Leesutthiphonchai and Judelson (2018b)
*PiBZP1*	bZIP transcription factor	Zoospore swimming behavior, cyst germination and aprpressorium formation, pathogenicity	Blanco and Judelson (2005)
*PlBZP32*	Per-ARNT-Sim (PAS)-containing bZIP transcription factor	Sporangium production, sporangiophore development, cyst germination, oxidative stress tolerance, extracellular peroxidases and laccases activity, pathogenicity	Kong et al. (2020)
*PiCdc14*	Dual-specificity protein phosphatase	Sporangium production and morphology	Ah-Fong and Judelson (2003)
*PsCdc14*	Dual-specificity protein phosphatase	Sporangium production	Zhao et al. (2011a)
*PsYKT6*	Soluble N-ethylmaleimide-sensitive factor attachment protein receptor	Mycelial growth and morphology, sporangium production, zoospore release oospore production, pathogenicity	Zhao et al. (2011b)
*PiCAT2*	Catalase	Mycelial growth, sporangium production and germ tube production, zoospore release, cyst germination, pathogenicity	Wang et al. (2009)
*PsATG6a*	Autophagy-related protein	Sporangium production, haustorial formation, pathogenicity	Chen et al. (2017)
*PlATG6a*	Autophagy-related protein	Mycelial growth, sporangium production, zoospore release, sporangiophore development, oxidative and salt stress tolerance, pathogenicity	Wang et al. (2022)
*PsGRASP*	Golgi reassembly stacking protein	Mycelial growth, zoospore release, oxidative and endoplasmic reticulum tolerance, extracellular laccases activity, pathogenicity	Si et al. (2021a)
*PsHint1*	Histidine triad (HIT) domain-containing protein	Zoospore chemotaxis and encystment, cyst germination and polarized growth, pathogenicity	Zhang et al. (2016)
*Pi-RNH1*	DEAD box RNA helicase	Zoospore development	Walker et al. (2008)
*PnDLC1*	Dynein light chain 1	Zoospore motility	Narayan et al. (2010)
*PnPMA1*	Plasma membrane H^+^-ATPase	Zoospore development and swimming behavior as well as encystment, cyst germination, pathogenicity	Zhang et al. (2012b)
*PpMID1*	Mating pheromone-induced death 1	Sporangium production, morphology and cleavage, pathogenicity	Hwu et al. (2017)
*PsIMPA1*	Importin α subunit	Mycelial growth, sporangia production, oospore production, oxidative stress tolerance, reactive oxygen species detoxification, pathogenicity	Yang et al. (2015)
*PiNIFC1*/*2*/*3*	Spore-specific nuclear LIM interactor-interacting factors	Cyst germination	Judelson and Tani (2007)
*PcCHS/PsCHS1*	chitin synthase	Mycelial growth, sporangia production, zoospore release, pathogenicity	Cheng et al. (2019)
*PcPdeH*	High-affinity cAMP phosphodiesterase	Mycelial growth, cyst germ tube polarized growth, oxidative stress tolerance, intracellular cAMP level, pathogenicity	Li et al. (2020a)
*PcFtsZ2*	FtsZ protein	Mycelial growth, sporangiophore development, sporangia production and morphology, pathogenicity	Li et al. (2020b)
*PsHSF1*	Heat shock transcription factor	Cyst germination，oxidative and heat shock stress tolerance，extracellular peroxidases and laccasesactivity，pathogenicity	Sheng et al. (2015)
*PsGPI16*	GPI transamidase	Cyst germination, pathogenicity	Zhang et al. (2021)
*PsHSP70*	Heat shock protein	Oospore production, pathogenicity	Zhang et al. (2021)
*PlM90*	Puf RNA-binding protein	Zoospore release and encystment, oospore production	Jiang et al. (2017)
*PuM90*	Puf RNA-binding protein	Oospore development	Feng et al. (2021)
*PuFLP*	Flavodoxin-like protein	Oospore development	Feng et al. (2021)
*PsCZF1*	C_2_H_2_ zinc finger protein	Mycelial growth, zoospore release, cyst germination, oospore production, pathogenicity	Wang et al. (2009)
*PlCZF1*	C_2_H_2_ zinc finger protein	Oospore development, extracellular laccases activity, pathogenicity	Zhu et al. (2022)
*PiLLP*	Loricrin-like protein	Mycelial growth, sporangia production, zoospore release, cyst germination, oospore development, oxidative stress tolerance, pathogenicity	Guo et al. (2017)

Distinction from other spores which are short-live and survive poorly apart from the host, the thick-walled oospores resulted from sexual reproduction are a multiyear threat because they can persist for years in soil, surviving freezing and fungicides. For example, in regions of northern Europe and Mexico where sexual reproduction of the heterothallic species *P. infestans* is frequent, oospores are an important source of inocula (Lehtinen and Hannukkala, 2004; Grüwald and Flier, 2005). Although mating type in *P. infestans* is determined by a single locus, duplication, transposition, deletion, or other rearrangement of this locus make the mating event complicated (Judelson, 1996). We anticipate that more research should be carried out in this direction to better seize the details of mating type determination in *Phytophthora* and *Peronophythora*. Modification of mating hormones or application of stimulants to alter oospore germination period might enable reduction of oospore infection.

Besides the *Phytophthora*, *P. litchii* is also an ideal research material, because the genome of this pathogen is published and it is easy to be genetically manipulated (Ye et al., 2016). *Phytophthora* and *Peronophythora* are the hemibiotrophic oomycetes which consist of biotrophic and necrotrophic phases during their infection. Although recent advance in phylogenetic analysis based on genome data have suggested that *P. litchii* (the only species in *Peronophythora*) belong to *Phythophtora* (Ye et al., 2016), this pathogen produces *Peronospora*-like sporangiophores and the differentiated, branched sporangiophores of *P. litchii* suggest a taxonomic affinity with downy mildews species (Figure 3). Moreover, the germ tubes in *P. litchii* oospores also have the characteristics of both *Peronospora* and *Phytophthora*, which comprise the short germ tubes that do not develop further and germ tubes that terminated by sporangia or become mycelia (Pao and Ko, 1980). Therefore, it is possible that *P. litchii* is the transitional species between *Phytophthora* and *Peronospora*. In future, this is important for elucidating the molecular mechanism underlying the sporangiophores formation of *P. litchii*.

Understanding the spore formation pathways might lead to develop novel effective strategies for controlling oomycete diseases. Cross-kingdom RNA trafficking opens a novel avenue to crop protection (Cai et al., 2018). The exchange of small RNAs (sRNAs) between hosts and downy mildew pathogen *Hyaloperonospora arabidopsidis* and the disease control have been reported (Bilir et al., 2019; Dunkerl et al., 2020). SIGS (Spray-induced gene silencing) for *P. infestans* control was also attempted, though low dsRNA uptake efficiency application of dsRNA fails to inhibit the virulence of this pathogen (Qiao et al., 2021). However, in a very recent research Sundaresha et al. developed multigene targeted dsRNA molecules, along with nanoclay carriers, to effectively reducing late blight infection (Sundaresha et al., 2022). These researches pave the way for designing artificial sRNA according to sporulation-related genes and applying SIGS or HIGS (Host-induced gene silencing) to control diseases caused by *Phytophthora* and *Peronophythora* pathogens.

## Author contributions

JS and GK contributed to the conception and design of the work. JS, PX, and GK acquired most of the information and wrote sections. All authors contributed to the article and approved the submitted version.

## Funding

The authors were supported by the Natural Science Foundation of Guangdong Province, China (2022A1515010458, 2020A1515011335, and 2019A1515010977), National Natural Science Foundation of China (31701771), the earmarked fund for CARS-32.

## Conflict of interest

The authors declare that the research was conducted in the absence of any commercial or financial relationships that could be construed as a potential conflict of interest.

## Publisher’s note

All claims expressed in this article are solely those of the authors and do not necessarily represent those of their affiliated organizations, or those of the publisher, the editors and the reviewers. Any product that may be evaluated in this article, or claim that may be made by its manufacturer, is not guaranteed or endorsed by the publisher.
